# Noninvasive assessment of core metastatic genes in lung adenocarcinoma: development of a predictive model integrating single-cell transcriptomics and radiomics

**DOI:** 10.3389/fonc.2026.1784914

**Published:** 2026-03-16

**Authors:** Shengqian Wu, Tao Hu, Zhikai Cao, Chengbin Lin, Shuo Huang, Yingxi Li, Keyun Zhu, Yao Tian, Jinxian He

**Affiliations:** 1 Department of Thoracic Surgery, The Affiliated LiHuiLi Hospital of Ningbo University, Ningbo, Zhejiang, China; 2 Health Science Center, Ningbo University, Ningbo, Zhejiang, China

**Keywords:** LUAD, PSMB5, PSMB7, radiomics, SCL16A3, tumor metastasis

## Abstract

**Background:**

Lung adenocarcinoma (LUAD) leads to death primarily due to its high metastatic potential. Risk assessment methodologies currently predicated on histopathological and imaging features possess a limited capacity to predict metastatic potential. Therefore, integrating single-cell transcriptomics and CT radiomics to identify key molecular drivers of metastasis and establishing a noninvasive imaging prediction model for LUAD is important.

**Methods:**

Bulk transcriptomic data and single-cell RNA sequencing (scRNA-seq) data were obtained from public database for analysis. Analytical tools (Seurat, inferCNV, Monocle, WGCNA, LASSO regression, GO/KEGG/GSEA, CellChat) were used for cellular profiling, trajectory analysis, gene identification, functional enrichment, and cell–cell communication. Immunohistochemistry (IHC) and RT-qPCR validated candidate genes at protein and mRNA levels. Additionally, a CT radiomics-based predictive model was developed for noninvasive gene expression assessment.

**Results:**

ScRNA-seq analysis revealed a malignant cellular trajectory from primary to metastatic LUAD and identified a metastasis-associated subpopulation. Three consistently overexpressed genes (PSMB5, PSMB7 and SLC16A3) were correlated with poor prognosis. Functional studies indicated their synergistic roles in promoting tumor progression through cell cycle regulation, proteasome activity, and metabolic reprogramming. A CT radiomics model effectively predicted the combined expression of these genes (AUC = 0.765), linking imaging features to molecular phenotypes.

**Conclusion:**

This study reveals that the synergistic expression pattern of PSMB5, PSMB7 and SLC16A3 is closely associated with lung adenocarcinoma metastasis and poor prognosis, confirming their potential value as prognostic biomarkers and therapeutic targets. The CT radiomics model offers a noninvasive tool for molecular phenotyping, aiding in preoperative precision assessment and advancing noninvasive clinical decision-making for LUAD.

## Introduction

Lung cancer is the most common type of cancer worldwide and the leading cause of cancer-related deaths, posing a major and persistent challenge to global public health (1–3). Histologically, lung cancer is chiefly classified into non-small cell lung cancer (NSCLC) and small cell lung cancer (SCLC), with NSCLC accounting for roughly 80% to 85% of all cases. Among the various subtypes of NSCLC, lung adenocarcinoma (LUAD) emerges as the most common. Clinically, metastasis is recognized as the principal factor leading to treatment failure and subsequent patient mortality in lung cancer; however, the underlying molecular mechanisms remain inadequately understood (4). Metastasis refers to the dissemination of tumor cells from the primary lung site to remote organs, including the brain, bones, lymph nodes, and liver (5). This intricate process is characterized by a multitude of factors, including dynamic interactions among diverse cell types within the tumor microenvironment, the regulatory influence of lung cancer stem cells, and pivotal biological events such as epithelial-mesenchymal transition (EMT), angiogenesis, and lymphangiogenesis. Tumor metastasis signifies a poorer prognosis (6) and remains notoriously difficult to predict. However, single-cell technologies hold the potential to address this challenge by identifying and utilizing markers specific to metastatic malignant epithelial cells, thereby enabling more accurate prediction of metastatic propensity.

The advent of single-cell omics technologies has significantly advanced life sciences research. These methods enable high-resolution molecular profiling at the individual cell level, facilitating the creation of comprehensive cellular atlases for tissues and diseases. In contrast to traditional bulk sequencing techniques, single-cell analysis simultaneously captures genetic and transcriptomic data, thereby elucidating gene expression networks that are specific to distinct cell types. Furthermore, it enables precise identification of cell subtypes and offers an in-depth exploration of intratumoral heterogeneity (7). In the field of oncology, single-cell technologies furnish novel insights into tumor heterogeneity, assist in pinpointing potential therapeutic targets, and aid in the identification of biomarkers predictive of disease progression (8). Notably, single-cell RNA sequencing can reveal cellular heterogeneity within the tumor microenvironment, differences which are often undetectable in bulk analyses (9). This capability significantly advances tumor biology research. By integrating multi-omics technologies, it provides crucial multidimensional insights for both cancer research and precision medicine.

In this study, we performed a systematic comparative analysis of primary LUAD samples and their matched metastatic lesions using single-cell RNA sequencing. This approach led to the identification of three key genes (PSMB5, PSMB7, and SLC16A3) that were significantly upregulated in metastatic sites. The expression levels of these genes showed a significant positive correlation with poor patient survival outcomes. By integrating multimodal imaging and bioinformatic analyses, we further confirmed a significant positive association between the expression of these genes and more aggressive tumor imaging phenotypes, thereby establishing a robust link between molecular expression and imaging characteristics. These findings suggest that the identified genes not only serve as potential biomarkers for LUAD metastasis but also provide a novel molecular imaging foundation for the early non-invasive assessment of metastatic risk.

## Materials and methods

### Data acquisition and processing

In this investigation, a comprehensive analysis was performed on a total of 830 samples, which included four single-cell RNA sequencing (scRNA-seq) datasets obtained from the Gene Expression Omnibus (GEO) database. Among these samples, two originated from primary LUAD cases, while the other two were derived from metastatic LUAD specimens. Furthermore, a validation cohort comprising 226 LUAD RNA-seq samples was sourced from the GSE31210 dataset, alongside 600 RNA-seq samples extracted from The Cancer Genome Atlas (TCGA) LUAD cohort, which included 59 normal tissue samples and 541 tumor samples. For the TCGA-LUAD cohort, gene expression profiles were obtained using the TCGA biolinks software package (10). All expression data were standardized by converting them into transcripts per million (TPM) formats, followed by log2 transformation. Quality control measures and subsequent bioinformatics analysis for the scRNA-seq data were executed using Seurat (v4.3.0). Quality control of single-cell data was performed to exclude low-quality cells, with the following inclusion criteria: (1) 200–7,000 detected genes per cell; (2) mitochondrial gene proportion < 20% of total genes. Cells failing to meet either criterion were discarded. Potential doublet cells were identified and removed employing the scDblFinder tool. Normalization, dimensionality reduction, and clustering were carried out following established methodologies. The annotation of cell types was performed using marker genes sourced from the CellMarker database (11).

### CNV and single-cell downstream analysis

We employed the gene expression matrices derived from scRNA-seq samples (GSM7509499, GSM7509500, GSM8528674, GSM8528675), designated as AIS1, AIS2, MT1, and MT2, respectively, to conduct an independent analysis of copy number variation (CNV) for each sample utilizing the inferCNV package. Immune cells, predominantly B cells, were established as the reference population, allowing for the inference of CNVs in epithelial cells through comparative analysis of their expression profiles against this reference cohort. Epithelial cells were classified as malignant based on the criteria of a CNV score exceeding 0.001 and a CNV correlation greater than 0.4. After the identification of malignant epithelial cells, differentiation trajectory analysis was performed using the Monocle 2 package (12). Initially, the expression profiles were transformed into a Monocle object, followed by processes of normalization and gene filtering. Non-linear dimensionality reduction was executed using the DDRTree algorithm (with a maximum of two dimensions) to delineate the cellular differentiation trajectory. To investigate the functional dynamics during differentiation, we applied Gene Set Variation Analysis (GSVA) (13) to evaluate the activity of hallmark pathways across distinct tumor subpopulations. Following this, functional enrichment analysis of the identified active pathways was conducted utilizing the clusterProfiler package (14). Finally, an analysis of clinical survival associations was performed for cell subpopulations exhibiting significant pathway enrichment and functional divergence.

### Analysis of cell-cell communication

The investigation of cell-cell communication within the microenvironment of LUAD was conducted utilizing the CellChat package (15). Malignant cells were categorized into primary and metastatic subgroups, with a particular emphasis on metastatic malignant cells as the primary subject of analysis, while other cell types were regarded as the background for communication. Prior to the analysis, the data underwent quality control measures, ensuring a minimum of 50 cells per group, alongside subsampling, which was implemented when any group exceeded 5000 cells. Employing the single-cell RNA sequencing count matrix in conjunction with the CellChatDB.human database (16), we identified genes that were overexpressed, along with ligand-receptor pairs present in each cell group. The probabilities of communication were computed with the trim parameter set to 0.1 to capture even weak signaling activities, and the resulting data were further refined using a threshold that required a minimum of five interacting cells. An aggregated network of cell-cell communication was established through the application of the aggregateNet function. The interaction patterns, as well as key signaling pathways, were illustrated using circle plots, heatmaps, and chord diagrams. Our analysis particularly concentrated on the functional roles of metastatic malignant cells, considering them as both signal senders and receivers within the communication network.

### High-dimensional weighted gene co-expression network analysis

In this study, we utilized the hdWGCNA package to establish gene co-expression networks specifically within epithelial cells (17). Following the clustering and annotation of cells via Seurat, the samples were categorized into primary and metastatic groups. To mitigate technical noise, metacells were created with a parameter of k = 25, and the resulting expression matrices were subsequently normalized. A weighted gene co-expression network was built with a soft-thresholding power of 8, resulting in the detection of five separate gene modules. These modules were defined by a minimum size of 10 genes and a merge cut height set at 0.10. For each identified module, we selected the top 50 genes, ranked according to their module membership (measured through eigengene-based connectivity) and gene significance, to serve as hub genes for further downstream analyses. Additionally, we assessed and visualized the network topology as well as the relationships between modules and traits, thereby providing insights into the overall architecture of the gene co-expression networks.

### Survival analysis and prognostic modeling

Integrated clinical and transcriptomic data from the TCGA-LUAD cohort served as the basis for prognostic modeling in this study. The Kaplan–Meier method, implemented with the survival package (18), was used for survival analysis, and differences between groups were evaluated using the log-rank test. Feature genes were selected and a risk score model was built via LASSO-Cox regression analysis using the glmnet package. To ensure the model’s robustness, ten-fold cross-validation was utilized. The visualization of risk scores, survival status, and the associated survival curves was accomplished with the survminer package (19). The model’s predictive performance was evaluated at 1-, 2-, and 3-year follow-up points using time-dependent receiver operating characteristic (ROC) analysis implemented with the timeROC package (20). Prior to the execution of these analyses, data cleaning and preprocessing were performed using the dplyr package (21) to uphold the quality and reproducibility of the analytical outcomes. Differentially expressed genes in malignant metastatic epithelial cells were screened using the FindAllMarkers function (Seurat) with the following criteria: min.pct = 0.3, log_2_FC > 0.5, and min.diff.pct = 0.2. Wilcoxon test was applied, and the top 100 genes with adjusted P < 0.01 were retained.

### Gene set enrichment analysis

Samples in the TCGA LUAD cohort were divided into high- and low-expression groups using the median expression level of the target gene set as the cutoff. Differential expression analysis was conducted with the limma package (22), applying linear modeling and empirical Bayes moderation to identify significant differentially expressed genes (adjusted *P*-value < 0.05). Following this, a comprehensive functional enrichment analysis was performed using the clusterProfiler package, which encompassed three fundamental analyses: 1) Gene Set Enrichment Analysis (GSEA) leveraging the MSigDB Hallmark gene sets (23); 2) enrichment analysis of Kyoto Encyclopedia of Genes and Genomes (KEGG) pathways; and 3) analysis of biological processes as per Gene Ontology (GO). For each of these analyses, the top 30 most significantly enriched terms or pathways were selected for detailed interpretation. Prior to the enrichment analysis, gene identifier conversion was executed via the org.Hs.eg.db database (24) to guarantee the precision and dependability of the analytical outcomes.

### Lung adenocarcinoma specimens

The diagnosis of all samples was confirmed by histopathological methods. The specimens were collected from the Affiliated LiHuiLi Hospital of Ningbo University. The study protocol received approval from the Ethics Committee of the Affiliated LiHuiLi Hospital of Ningbo University and was conducted in accordance with the ethical principles outlined in the Declaration of Helsinki. All participating patients provided written informed consent.

### Immunohistochemical assays

Paraffin-embedded tissue sections were sequentially subjected to deparaffinization, rehydration, antigen heat retrieval, endogenous peroxidase blocking, and non-specific serum blocking. The processed sections were subsequently incubated with respective primary antibodies overnight at 4 °C (antibody information is available in Supplementary File S1). On the following day, the sections were treated with horseradish peroxidase -conjugated secondary antibodies at room temperature. Target protein expression was then visualized using a DAB chromogenic kit (ZSGB-BIO, China) for signal development.

### Reverse transcription quantitative PCR

Total RNA was isolated from cells with TRIzol reagent (Invitrogen, USA) according to the manufacturer’s protocol, followed by cDNA synthesis using a reverse transcription kit (TaKaRa, Japan). The qPCR reaction mixture included 2 µL of cDNA template, sequence-specific primers, and SYBR Green PCR master mix (TaKaRa, Japan). Sequence-specific primers for PSMB5, PSMB7, SLC16A3 and GAPDH were synthesized by Genewiz (Tianjin, China). Their corresponding primer sequences are provided in Supplementary File S2.

### Computational drug repurposing screening

Potential compounds targeting the PSMB5/PSMB7/SLC16A3 network were screened using the Clue.io online platform (https://clue.io). The combined gene expression signature of PSMB5, PSMB7, and SLC16A3 were employed as the query signature. The platform compares this signature against the CMap database of drug-induced gene expression profiles and computing connectivity scores. Compounds with negative connectivity scores are predicted to induce effects opposite to the query signature. Top-ranking compounds with the most negative scores were selected for further mechanistic categorization and analysis.

### Extraction of radiomic features and development of predictive models

In the context of the TCGA LUAD cohort, we combined transcriptomic data with radiomic features. The three-dimensional reconstruction of imaging data and the extraction of features were executed utilizing 3D Slicer software (25), adhering to the protocols set forth by the Image Biomarker Standardization Initiative (IBSI). Tumor regions were meticulously outlined by a seasoned radiologist and subsequently confirmed by an expert reviewer. Prior to the extraction of features, all imaging data were uniformly resampled to achieve a consistent spatial resolution. Initially, a total of 129 valid radiomic features were extracted and subsequently normalized using Z-score transformation. Key feature selection was conducted through Pearson correlation analysis (26), resulting in the retention of 10 core features for the subsequent model development. A LASSO regression model was constructed employing the glmnet package, with the optimal regularization parameter (λ) being ascertained via ten-fold cross-validation. The predictive model was trained on a subset comprising 27 samples. Given the small sample size of the training cohort, a rigorous 3-fold cross-validation (27) was additionally performed to further validate the model’s reliability and mitigate the risk of overfitting. The performance of the model was assessed through receiver operating characteristic (ROC) curve analysis, utilizing the pROC package, and the predictive efficacy was quantified by calculating the area under the curve (AUC). Additionally, we further validated the model’s performance through correlation analysis executed with the stats package.

### Statistical analysis

Statistical analyses in this study were performed using R software (version 4.3.2). Differential gene expression analysis was conducted with the limma package, using an adjusted *P*-value (adj. *P*.Val) < 0.05 as the significance threshold. Survival analysis was carried out with the survival package, where Kaplan–Meier curves were generated and compared using the log-rank test; hazard ratios (HR) and 95% confidence intervals (CI) were derived from Cox proportional hazards models. Correlations between continuous variables were assessed using Pearson’s correlation coefficient. Group comparisons were selected based on data distribution: normally distributed data were analyzed with the t-test or ANOVA, while non-normally distributed data were analyzed using the Mann-Whitney U test or Kruskal–Wallis test. Categorical variables were compared with the chi-square test or Fisher’s exact test. The discriminative performance of prognostic and radiomics models was evaluated via receiver operating characteristic (ROC) curves and the corresponding area under the curve (AUC). To control the false positive rate in multiple hypothesis testing, the Benjamini-Hochberg procedure was applied to adjust P-values and calculate the false discovery rate (FDR). Data visualization was primarily implemented using packages such as ggplot2, pheatmap, survminer, and Complex Heatmap. Unless otherwise noted, all statistical tests were two-sided, with significance levels set at *P < 0.05, **P < 0.01, and ***P < 0.001.

## Results

### Identification of LUAD cell types using single-cell RNA sequencing

**Figure 1:**
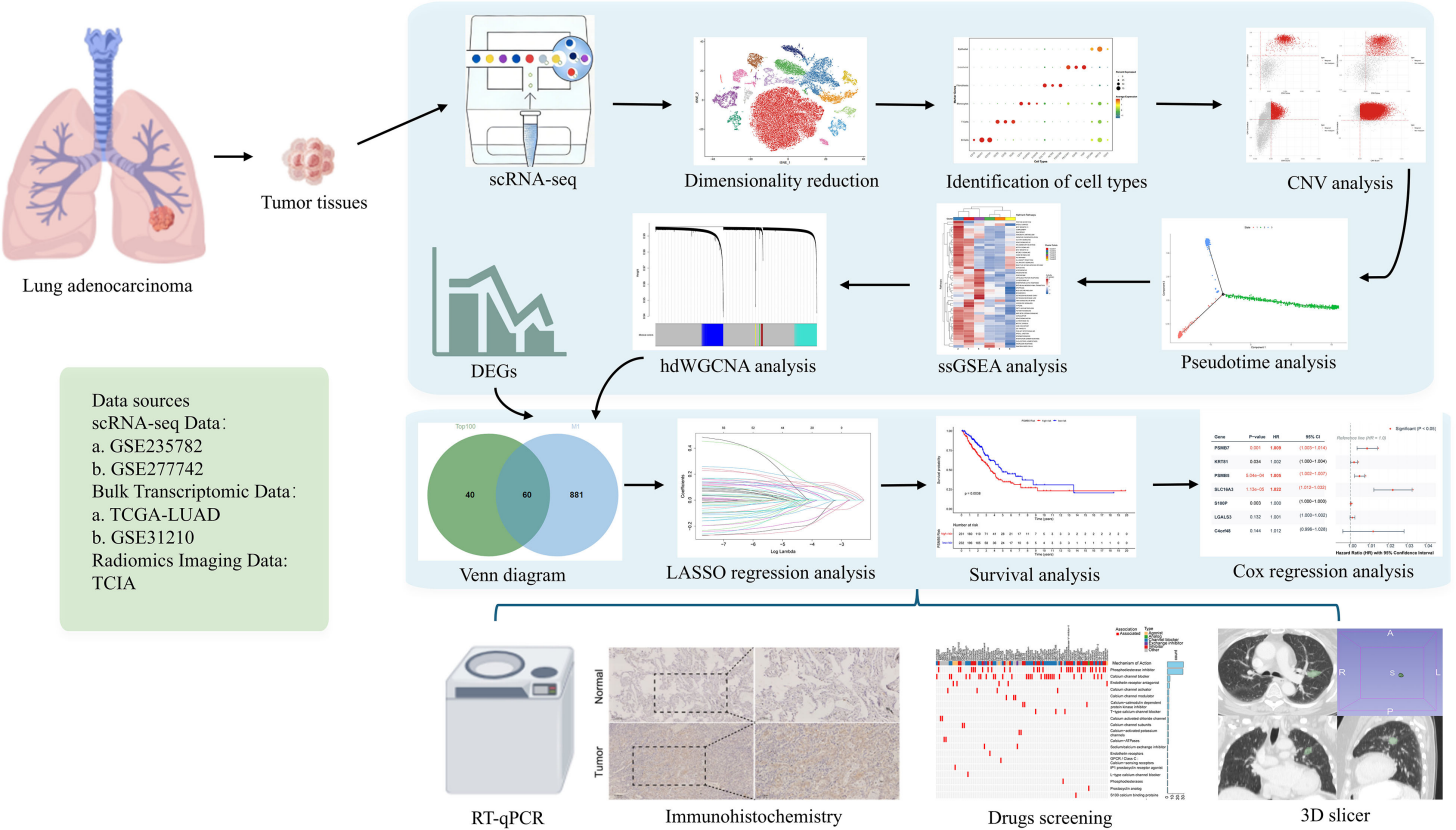
Overall study workflow diagram. GSE, Gene Expression Omnibus Series; TCGA, The Cancer Genome Atlas; TCIA, The Cancer Imaging Archive; DEGs, Differentially Expressed Genes.

**Figure 2:**
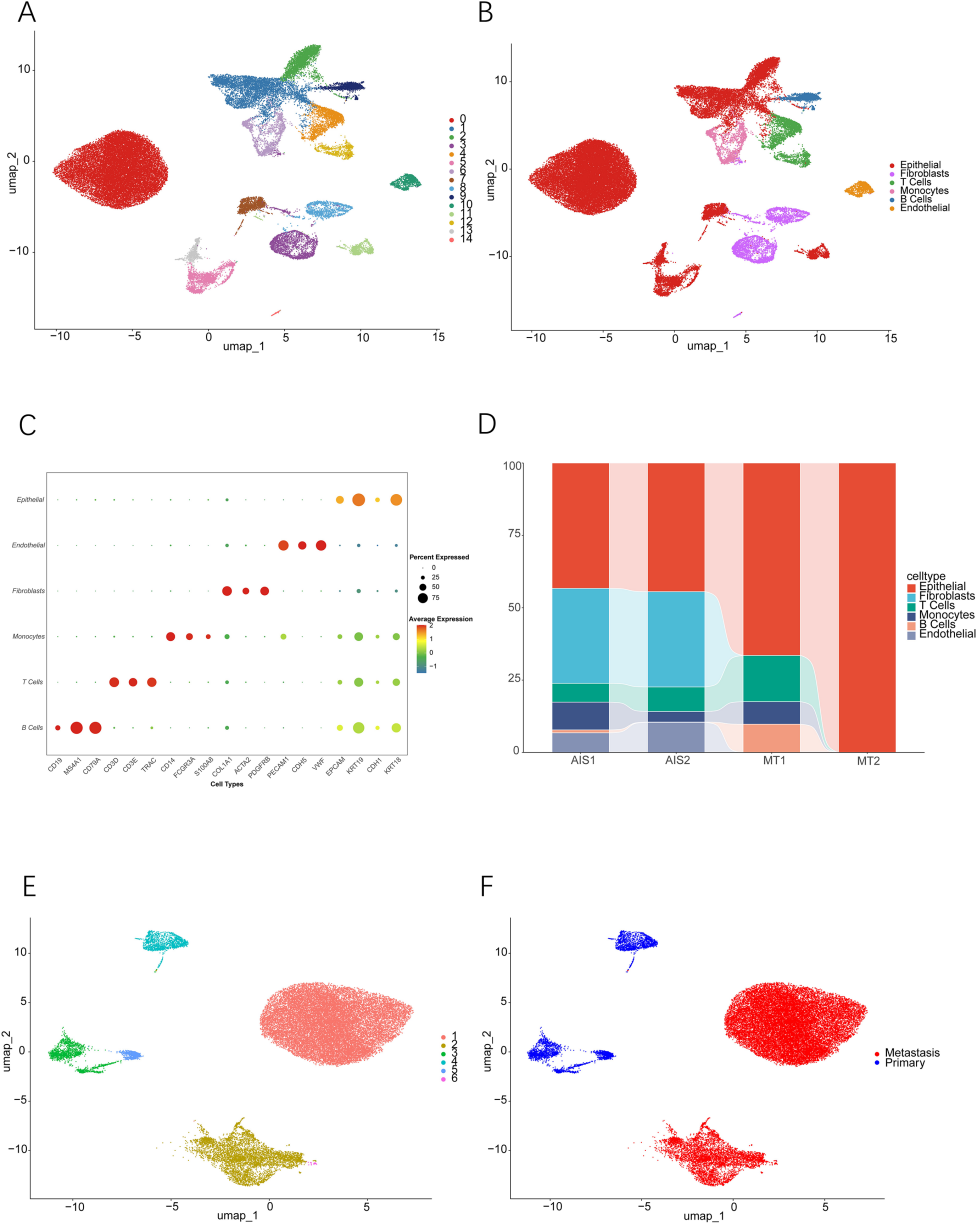
Construction of the lung adenocarcinoma cellular atlas based on single-cell sequencing and copy number variation inference analysis of malignant epithelial cells. **(A)** UMAP plot displaying 15 distinct cell clusters within LUAD samples. **(B)** Dot plot illustrating the expression of marker genes used to annotate the six maillustrateses. **(C)** UMAP visualization depicting the spatial distribution of the six annotated cell types across LUAD samples. **(D)** Proportional composition of each cell type across the analyzed samples. **(E)** Distribution of the six malignant epithelial cell subpopulations visualized in UMAP space. **(F)** Comparative UMAP analysis between primary and metastatic malignant epithelial cells.

The comprehensive workflow employed in this investigation is illustrated in **Figure 1**. Following rigorous quality control utilizing the Seurat pipeline, a total of 46,274 high-quality cells were preserved, comprising 11,803 cells derived from primary LUAD samples (AIS1 and AIS2) and 34,471 cells sourced from lymph node metastatic samples (MT1 and MT2). Utilizing principal component analysis with a resolution parameter set at 0.2, the entire scRNA-seq dataset was categorized into 15 distinct clusters. Visualization through graph-based dimensionality reduction revealed a pronounced spatial segregation among the various cell clusters (**Figure 2A** and **Supplementary Figure 1A**). Subsequently, all cells were annotated with marker genes obtained from the CellMarker database (**Figure 2C**), enabling the identification of six predominant cell types: epithelial cells, fibroblasts, T cells, monocytes, endothelial cells, and B/plasma cells (**Figure 2B** and **Supplementary Figure 1B**). This annotation strategy effectively distinguished between diverse cell lineages. For instance, seven clusters (0, 1, 2, 5, 7, 11 and 13) were classified as epithelial cells based on the expression of specific marker genes, including EPCAM, KRT19, and CDH1. The proportional representation of these six cell types across all LUAD samples is illustrated in **Figure 2D**. In summary, we successfully delineated six major cell populations, thereby facilitating subsequent analytical endeavors.

### Identification of malignant cells in LUAD through InferCNV analysis

To distinguish between benign and malignant epithelial cells at the single-cell level, we performed inferCNV analysis combined with a dual-threshold approach to identify malignant cells. Utilizing B cells as a reference population, we computed two critical metrics for each epithelial cell: the CNV score (which quantifies the extent of copy number variation) and CNV correlation (which evaluates the similarity to a canonical malignant genomic profile). Epithelial cells meeting both criteria, namely a CNV score above 0.001 and a CNV correlation over 0.4, were designated as malignant. As illustrated in **Supplementary Figures 1C–F**, the malignant cells identified through inferCNV exhibited significant transcriptional heterogeneity and were distinctly separated from the reference B cells as well as other non-malignant cell populations. The spatial distribution of malignant epithelial cells in relation to other cell types is depicted in **Supplementary Figures 2A, B**. For subsequent analysis, we initially excluded non-epithelial lineages and retained only epithelial cells to distinguish between normal and malignant epithelial cells (as shown in **Supplementary Figure 2C**). Subsequently, normal epithelial cells were removed, ultimately forming a cohort of 30,563 malignant epithelial cells (as shown in **Supplementary Figure 2D**). Following dimensionality reduction and clustering at a resolution of 0.2, we identified six distinct malignant subpopulations (UMAP in **Figure 2E**; t-SNE in **Supplementary Figure 2E**). These subpopulations were further categorized into primary or metastatic malignant epithelial cells based on their tissue of origin (UMAP in **Figure 2F**; t-SNE in **Supplementary Figure 2F**). In summary, we successfully isolated malignant epithelial cells for further functional analysis.

### Cellular trajectory and functional diversity of malignant subpopulations in lung adenocarcinoma

**Figure 3:**
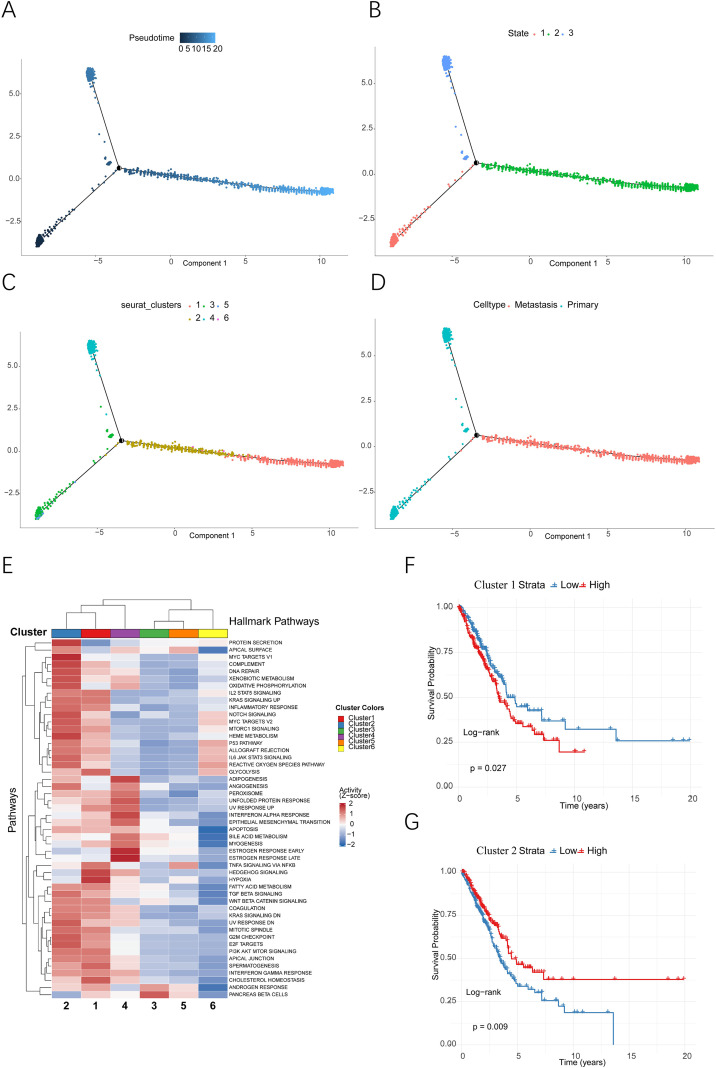
Developmental trajectory, molecular characteristics and clinical prognosis analysis of malignant epithelial cell subpopulations in lung adenocarcinoma. **(A)** Pseudotime trajectory analysis based on single-cell transcriptomics illustrates the developmental trajectory of malignant epithelial cells. **(B)** Distribution of cells with distinct transcriptional states across the developmental trajectory. **(C)** Localization of six malignant epithelial cell subpopulations along the developmental trajectory. **(D)** Distribution of cells from primary and metastatic sites along the developmental trajectory. **(E)** Heatmap of Hallmark pathway enrichment based on GSEA analysis across tumor subpopulations. **(F, G)** Kaplan-Meier survival curve analysis demonstrating the correlation between the abundance of Cluster 1 and Cluster 2 subpopulations and overall survival in lung adenocarcinoma patients. Statistical significance was assessed using the two-sided log-rank test for survival comparisons, and hazard ratios (HR) with 95% confidence intervals (CI) were estimated using univariate Cox proportional hazards regression.

Transcriptional variability throughout tumor progression represents a defining characteristic of the metastatic evolution observed in LUAD. To elucidate the dynamic evolutionary trajectory of malignant epithelial cells, this study constructed a cell differentiation pathway map. The analysis revealed three distinct cellular states (**Figure 3A**) and three independent evolutionary branches (**Figure 3B**), with the initial state (State 1) representing an early cell population within the malignant progression continuum. Further analysis of the trajectory indicated that cell clusters 5 and 3 transitioned sequentially into clusters 2, 6, and 1 along the developmental continuum (**Figure 3C**). This transition corresponds to the phenotypic shift from primary malignant cells to cells exhibiting metastatic characteristics (**Figure 3D**), highlighting the differentiation process from primary tumor cells to metastatic tumor cells in LUAD. The dynamic expression patterns of differentially expressed genes throughout the evolutionary trajectory are illustrated in the heatmap (**Supplementary Figure 3A**). To examine the functional diversity among malignant subpopulations, we assessed pathway activity utilizing Hallmark gene sets. Pathways linked to malignant tumors, including E2F and PI3K signaling, showed significant enrichment in both cluster 1 and cluster 2 (**Figure 3E**). We further performed survival analysis on clusters 1 and 2, which revealed a significant association between cluster 1 and poor patient prognosis (**Figures 3F, G**). These results imply that these two subpopulations are metastasis-associated subpopulations. In summary, our analyses highlight considerable functional diversity throughout the evolution of malignant LUAD cells, chart a distinct developmental pathway from primary to metastatic states, and provide novel insights into the molecular mechanisms that drive metastasis in LUAD.

### Single-cell analysis uncovers cell-cell communication networks

In order to elucidate the intricate dynamics between intercellular communication and transitions in cellular states throughout the progression of LUAD, we conducted a thorough analysis of cell-cell interaction networks within the tumor microenvironment utilizing CellChat, informed by scRNA-seq dataset. Attention was directed towards delineating the communication profiles of metastatic malignant subpopulations. Our findings indicated that metastatic cells assume a pivotal regulatory role within the broader cell-cell communication network. This cell population engages in extensive and vigorous interactions with stromal cells and immune cells (**Supplementary Figures 3B, C**). At the level of signaling pathways, metastatic cells predominantly function as principal signal senders through the macrophage migration inhibitory factor (MIF) pathway (**Supplementary Figure 3D**), thereby facilitating the remodeling of the tumor microenvironment. Concurrently, they exploit the mitogen-activated protein kinase (MK) pathway (**Supplementary Figure 3E**) to link various stages of tumor evolution between primary and metastatic sites, underscoring the critical role of this pathway in cross-stage tumor progression. Further quantitative assessments revealed that, when serving as signal senders (**Supplementary Figure 4A**), metastatic cells exert significant regulatory influences on monocytes and T lymphocytes. This observation suggests their potential involvement in modulating immune cell functionality to promote immune evasion or drive inflammatory responses. Conversely, when acting as signal receivers (**Supplementary Figure 4B**), these cells predominantly absorb signals from fibroblasts, highlighting the essential role of stromal cells in fostering the adaptation and survival of metastatic cells. In summary, from the perspective of cellular interactions, our research illustrates that metastatic LUAD cells can effectively remodel the tumor microenvironment through the coordination of multiple pathways and intense intercellular communication, thereby establishing a pro-metastatic niche that propels malignant progression. To investigate how malignant epithelial subpopulations interact with the tumor microenvironment in lung adenocarcinoma, we performed cell–cell communication analysis on the six malignant clusters. Preliminary findings (**Supplementary Figures 4C, D**) showed that Cluster 1 exhibited high centrality in the communication network, suggesting it may serve as a key hub. **Supplementary Figure 4E** revealed that the MIF and MK pathways are potentially involved in regulating myeloid immunity and angiogenesis, with clusters C1, C2, and C6 showing notably stronger signaling toward monocytes and endothelial cells. These insights offer valuable clues for unraveling the molecular mechanisms that underpin LUAD metastasis.

### Detection of co-expressed gene modules in LUAD cells

**Figure 4:**
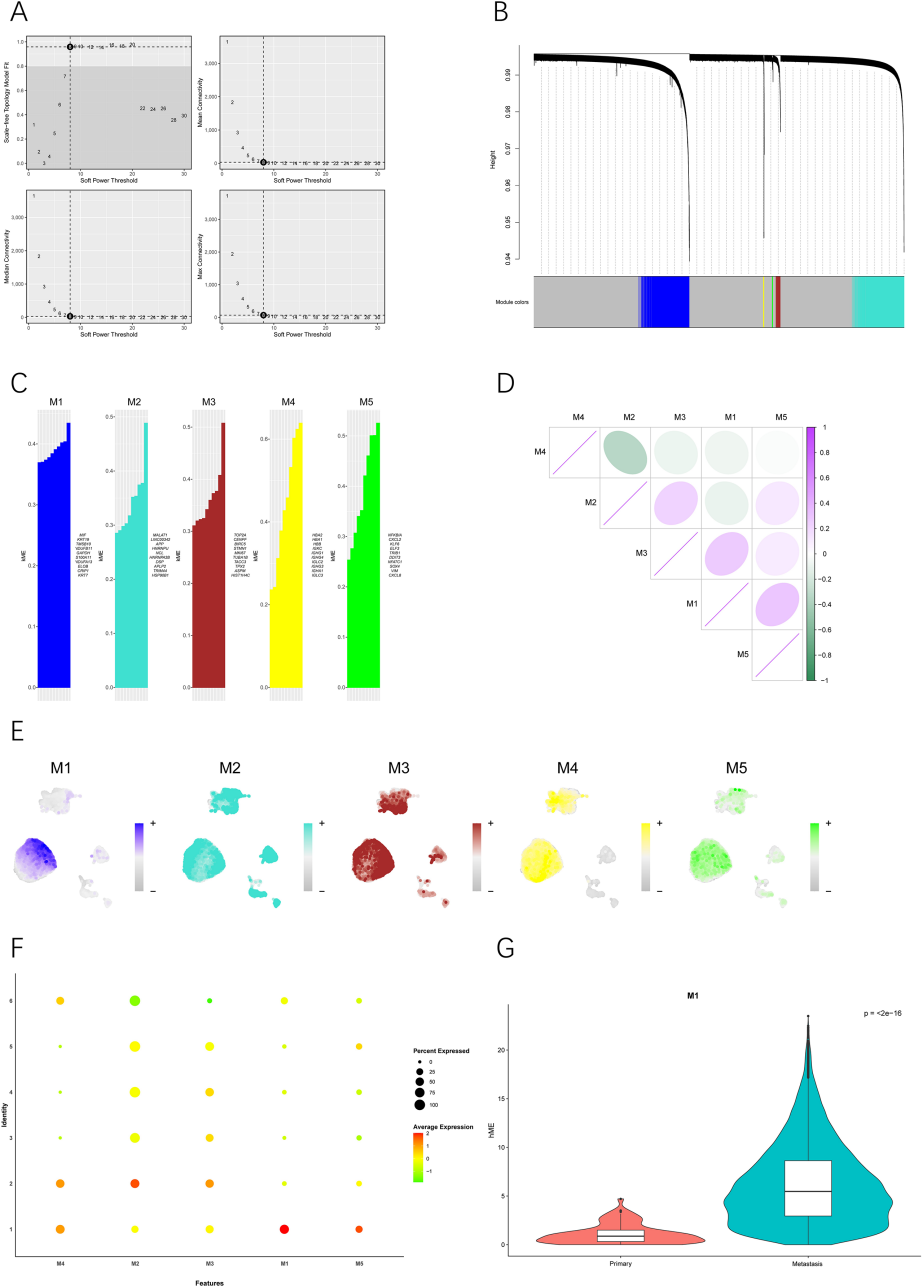
Identification and Analysis of Gene Co-expression Modules in Malignant Epithelial Cells of Lung Adenocarcinoma. **(A)** Schematic representation of the gene co-expression network topology constructed by Weighted Gene Co-expression Network Analysis (WGCNA). **(B)** Hierarchical clustering dendrogram of the five gene co-expression modules identified through dynamic tree cutting. **(C)** List of the top 10 hub genes ranked by connectivity within each module. **(D)** Heatmap illustrating the correlation of gene expression among different co-expression modules. **(E)** UMAP visualization of modules 1–5 across all malignant cells. **(F)** Dot plot demonstrating the strength of associations between each module and malignant epithelial cell subpopulations. **(G)** Comparison of expression scores of Module 1 in metastatic versus primary lung adenocarcinoma samples. Statistical significance was assessed using the two-sided Wilcoxon rank-sum test.

Based on the results from Section 3.3, the differentially expressed genes in Cluster 1 malignant epithelial cells were enriched in tumor progression-related pathways and showed a significant association with poor prognosis in LUAD patients. Therefore, it is essential to explore the co-expression gene network that plays a critical role within this subpopulation. To enhance connectivity, a scale-free gene co-expression network was built by applying the optimal soft-threshold power (β = 8). This network was confirmed to possess optimized connectivity features (**Figures 4A, B**). Analysis of this network revealed five distinct functional modules, highlighting the top ten most significant genes within each module (**Figure 4C** and **4E**). Inter-module correlation analysis demonstrated robust positive relationships among modules 1, 3, and 5, while the remaining two modules exhibited relative independence from this group (**Figure 4D**). Examination of the expression patterns of these modules across all cell clusters revealed that module 1 had the highest expression level specifically in cluster 1 (**Figure 4F**). Additionally, a significant difference in module 1 expression was noted between primary and metastatic tumor groups, with markedly higher levels found in metastatic samples (**Figure 4G**). Among the six malignant epithelial cell clusters, module 1 also showed the most distinct expression profile specific to cluster 1 (**Supplementary Figure 5A**). In summary, these findings suggest that module 1 represents a unique and highly active co-expression network specific to cluster 1, indicating its potential association with LUAD metastasis.

### Systematic identification of three key genes associated with LUAD metastasis and prognosis

**Figure 5:**
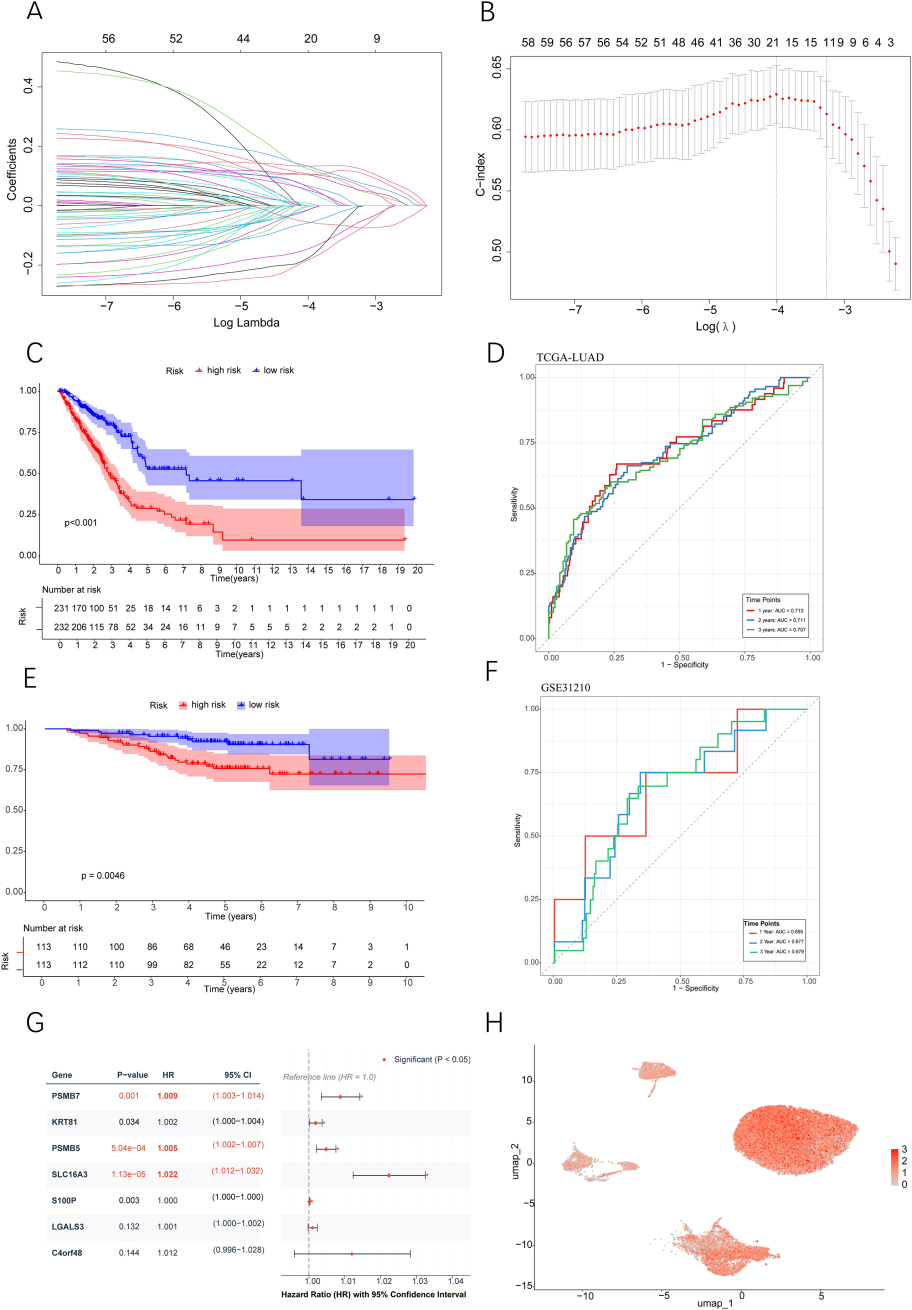
Screening and Prognostic Validation of Metastasis-Associated Gene Signatures in Lung Adenocarcinoma. **(A)** Coefficient variation path plot of LASSO regression analysis. **(B)** Error curve of ten-fold cross-validation in LASSO regression. **(C)** Kaplan-Meier curve of overall survival based on the 20-gene signature risk score in the TCGA-LUAD cohort. **(D)** ROC curve of the 20-gene signature for predicting patient survival in the TCGA-LUAD cohort. **(E)** Kaplan-Meier survival curve based on the same risk score in the independent GSE31210 cohort. **(F)** ROC curve of the 20-gene signature for predicting prognosis in the GSE31210 cohort. **(G)** Forest plot from multivariate Cox regression evaluating the independent prognostic value of key genes. **(H)** Expression distribution of PSMB5, PSMB7 and SLC16A3 in the single-cell transcriptomic UMAP space. Statistical significance was assessed using the two-sided log-rank test and univariate Cox regression for survival analysis, bootstrap resampling (1,000 iterations) for AUC 95% CI, and the two-sided Wald test in multivariate Cox regression for independent prognostic factors.

**Figure 6:**
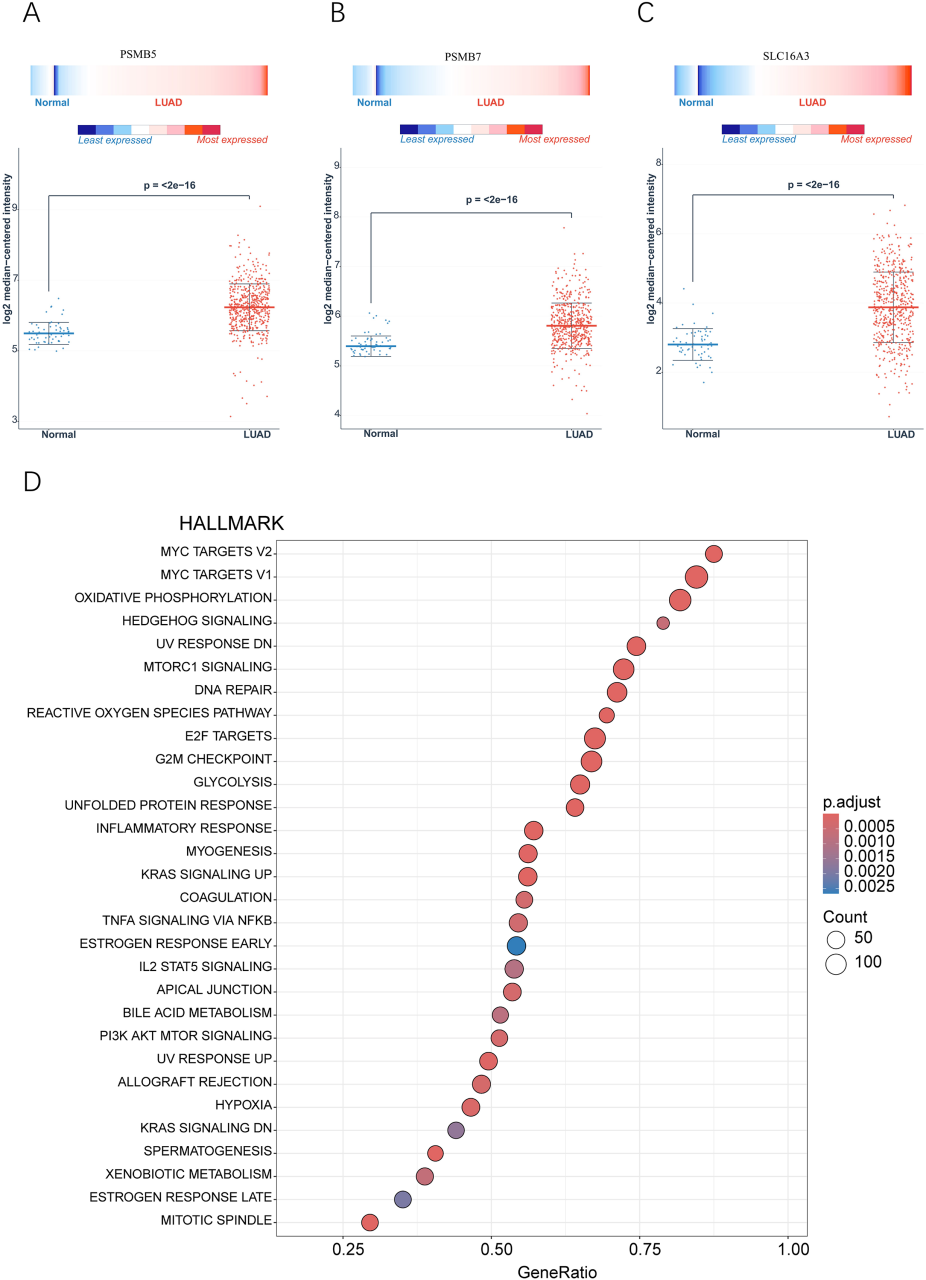
Flowchart of Prognostic and Expression Characteristic Analysis for Key Genes in Lung Adenocarcinoma. **(A-C)** Comparison of expression levels of PSMB5, PSMB7 and SLC16A3 between TCGA lung adenocarcinoma tissues and adjacent normal tissues. **(D)** GSEA enrichment analysis results based on the co-expressed gene set of PSMB5, PSMB7 and SLC16A3.

**Figure 7:**
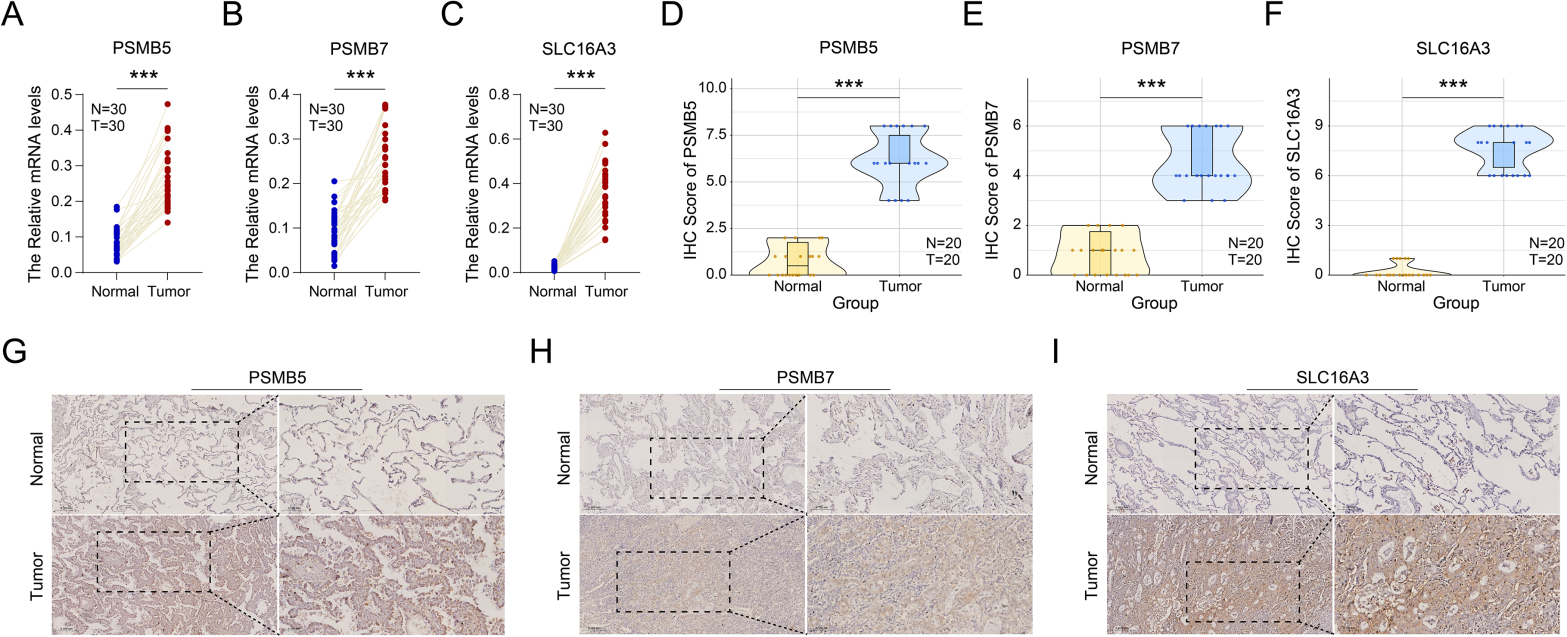
Validation of PSMB5, PSMB7, and SLC16A3 expression in paired LUAD clinical samples. **(A-C)** The mRNA expression levels of PSMB5 **(A)**, PSMB7 **(B)** and SLC16A3 **(C)** in 30 paired LUAD and adjacent normal tissues were determined by RT-qPCR. **(D-F)** IHC score of PSMB5 **(D)**, PSMB7 **(E)** and SLC16A3 **(F)** protein expression from IHC analysis in 20 matched LUAD and normal tissue pairs. **(G-I)** Representative immunohistochemistry (IHC) images showing the protein expression levels of PSMB5 **(G)**, PSMB7 **(H)** and SLC16A3 **(I)** in the same set of 20 paired LUAD and normal tissues. Statistical significance was assessed using the two-sided Wilcoxon signed-rank test for paired samples. ***P<0.001.

To systematically identify pivotal molecules that contribute to the metastasis of LUAD, we established a comprehensive multi-step gene screening protocol. Initially, we analyzed the transcriptomic profiles of tumor cells derived from both primary and metastatic lesions utilizing scRNA-seq data. This analysis revealed 100 significantly upregulated genes in metastatic tumor cells (Supplementary File S3). We subsequently cross-referenced this gene list with genes from co-expression module 1 (Supplementary File S4), resulting in a refined set of 60 candidate genes (as detailed in Supplementary File S5). Following this, we developed a survival prediction model utilizing the TCGA-LUAD cohort (**Supplementary Figures 5B, C**, **5A–C**). Through LASSO regression analysis, we further reduced the candidate genes to 20 that exhibited significant prognostic correlations (Supplementary File S6). The robustness of the prognostic predictive capability of this 20-gene set was confirmed via ROC curve analysis (**Figure 5D**), and this finding was subsequently corroborated in the independent GSE31210 cohort (**Supplementary Figures 5D, E**; **5E, F**). Subsequent stratification using the median expression levels of these genes (**Supplementary Figures 6A–G**) highlighted seven potential target genes: C4orf48, LGALS3, KRT81, PSMB7, PSMB5, S100P, and SLC16A3. Ultimately, multivariate Cox regression analysis (**Figure 5G**) validated three genes (PSMB5, PSMB7 and SLC16A3) as statistically significant prognostic indicators, supported by valid *P*-values, hazard ratios, and 95% confidence intervals. Validation studies indicated that the expression levels of these three genes were significantly higher in tumor tissues than in adjacent normal tissues in the TCGA-LUAD dataset (**Figures 6A–C**). Additionally, scRNA-seq data substantiated their extensive overexpression in LUAD (**Figure 5H**). Furthermore, we conducted validation on paired clinical samples using RT-qPCR and immunohistochemistry (IHC). RT-qPCR performed on 30 paired LUAD and adjacent normal tissue samples confirmed that PSMB5, PSMB7, and SLC16A3 mRNA levels were significantly elevated in tumor tissues relative to normal lung tissues (**Figures 7A–C**). In addition, IHC analysis of 20 matched LUAD and normal tissue pairs confirmed the significant upregulation of these three genes at the protein level in LUAD tissues (**Figures 7D–I**). Collectively, these findings suggest that the heightened expression of PSMB5, PSMB7 and SLC16A3 is intricately linked to LUAD metastasis and unfavorable patient prognosis. The consistent high expression of these genes in tumor tissues indicates their potential critical involvement in the malignant progression of LUAD.

### Multi-layered enrichment analysis identifies PSMB5, PSMB7, and SLC16A3 as synergistic drivers of LUAD malignancy

Integrated multi-omics analyses have demonstrated that PSMB5, PSMB7, and SLC16A3 function synergistically to promote the malignant progression of LUAD through multiple interconnected pathways. GO enrichment analysis (**Supplementary Figure 7A**) revealed that genes associated with these three key genes were significantly enriched in biological processes such as DNA replication, mitochondrial gene expression, and ribosome biogenesis. This finding implies a coordinated regulatory role in cell proliferation, energy metabolism, and protein synthesis. Furthermore, the KEGG pathway analysis (**Supplementary Figure 7B**) revealed substantial enrichment in pathways pertinent to the cell cycle, DNA replication, and proteasome functionality, underscoring their contributions to tumorigenesis through the modulation of cell proliferation and protein homeostasis. The GSEA results (**Figure 6D**) provided additional clarity by demonstrating that these three genes collectively augment cellular proliferative capacity via the activation of E2F target genes, the G2M checkpoint, and MYC signaling pathways (**Supplementary Figure 5F**). They also facilitate growth signal transduction through the mTORC1 and PI3K-AKT-mTOR pathways (**Supplementary Figure 5G**), induce metabolic reprogramming by simultaneously activating oxidative phosphorylation and glycolysis, and influence the tumor microenvironment by modulating hypoxia response and DNA repair pathways (**Supplementary Figure 5H**). Collectively, these results suggest that PSMB5, PSMB7, and SLC16A3 collaboratively enhance LUAD proliferation and metastasis through synergistic regulation of cell cycle dynamics, growth signaling, metabolic adaptation, and remodeling of the tumor microenvironment.

### Development of personalized treatment strategies for LUAD based on the synergistic axis of PSMB5, PSMB7, and SLC16A3

**Figure 8:**
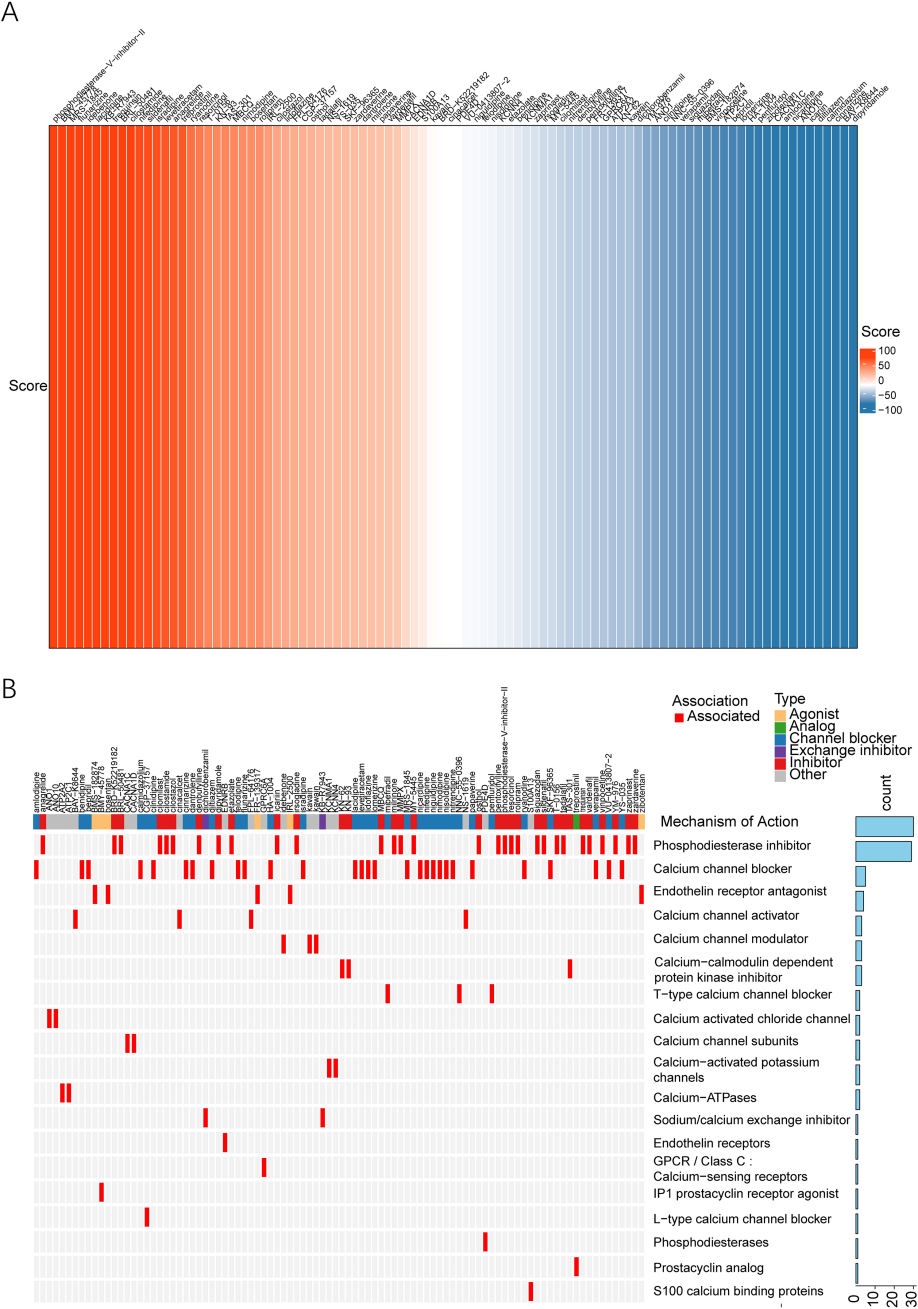
Investigation of potential therapeutic strategies involving combined targeting of PSMB5, PSMB7 and SLC16A3. **(A)** Screening workflow of potential therapeutic compounds and heatmap of inhibitory activity scores of candidate compounds against the PSMB5/PSMB7-SLC16A3 synergistic network. **(B)** OncoPrint classification of the mechanisms of action of candidate compounds: illustrating the distribution characteristics of mechanisms of action for different categories of compounds such as phosphodiesterase inhibitors and calcium channel blockers.

In response to the widespread challenges of targeted therapy resistance and chemotherapy resistance in current lung adenocarcinoma treatment, this study aims to explore innovative therapeutic strategies based on novel synergistic target combinations. A comprehensive screening of compounds was conducted utilizing the Clue.io online database, resulting in the identification of 94 potential targeted agents (Supplementary File S7). Among these candidates, several compounds demonstrated notable inhibitory effects against the synergistic regulatory network established by the three core genes; particularly, Phosphodiesterase-V-Inhibitor-II and KB-R7943 exhibited promising inhibitory efficacy (illustrated in **Figure 8A**). Notably, KB-R7943, an inhibitor of the Na^+^/Ca²^+^exchanger, has been reported in multiple tumor models to regulate intracellular ion homeostasis, thereby affecting tumor cell metabolism and survival (28, 29). Our results underscore its substantial potential to hinder the synergistic network involving PSMB5, PSMB7, and SLC16A3 in LUAD, suggesting that this compound may impede the malignant progression of tumors by disrupting the ionic equilibrium within cells. Mechanistically, the candidate compounds primarily operate through two pivotal pathways: the regulation of proteasome function and metabolic reprogramming. Notably, inhibitors of phosphodiesterase and ion exchange exhibit multi-target synergistic inhibition characteristics, which are consistent with the regulatory properties of the core gene axis. Further analysis using OncoPrint revealed that phosphodiesterase inhibitors (30 compounds) and calcium channel blockers (29 compounds) represent the two predominant mechanistic categories (as shown in **Figure 8B**). These agents are likely to exert anti-tumor effects by simultaneously targeting proteasome activity and cellular metabolism, thereby cooperatively inhibiting the pro-metastatic function mediated by PSMB5, PSMB7, and SLC16A3. In summary, this study identifies novel candidate compounds for the treatment of LUAD and offers mechanistic insights into multi-target synergistic approaches, establishing a theoretical basis for future experimental validation and translational research.

### Development of a CT radiomics-based model for noninvasive evaluation of PSMB5, PSMB7 and SLC16A3 expression in lung adenocarcinoma

**Figure 9:**
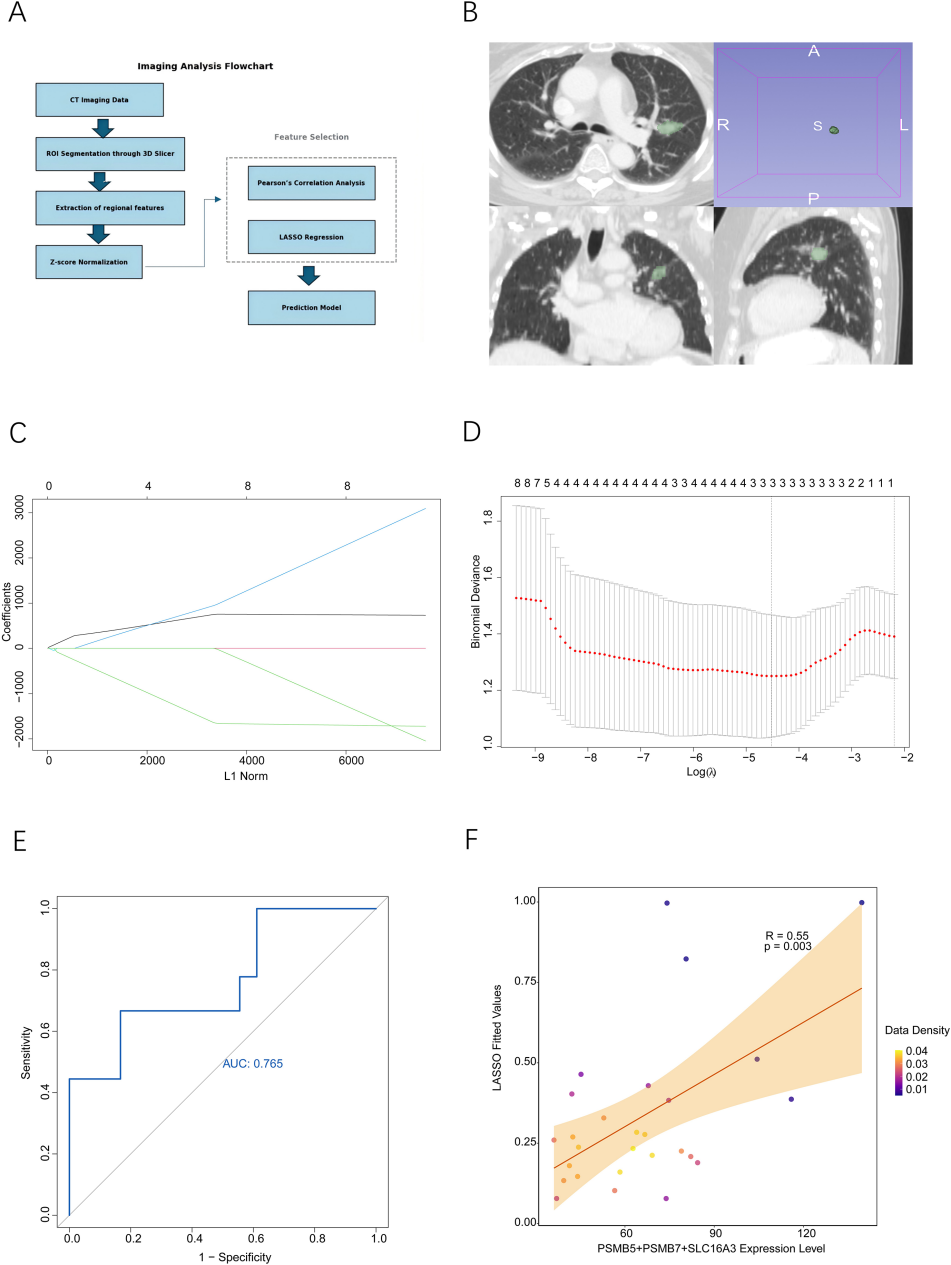
Construction and validation of a non-invasive gene expression assessment model based on CT radiomics. **(A)** Schematic diagram of the overall design and analytical workflow for CT radiomics research. **(B)** Example of tumor region (ROI) delineation on CT images. **(C)** LASSO regression coefficient path plot for radiomic feature selection. **(D)** Mean squared error curve of ten-fold cross-validation in LASSO regression. **(E)** ROC curve of the radiomics model for predicting gene expression status. **(F)** Scatter plot showing the correlation between model-predicted values and the aggregated expression of PSMB5, PSMB7 and SLC16A3. Statistical significance was assessed using bootstrap resampling (1,000 iterations) for AUC 95% CI and the two-sided Pearson correlation test for correlation analyses.

To advance the non-invasive evaluation of molecular phenotypes in LUAD, this study developed a CT-based radiomics model, aiming to provide a non-invasive auxiliary diagnostic tool for clinical practice (the specific workflow is shown in **Figure 9A**). For the construction of the model, a cohort of 27 LUAD patients was sourced from the TCIA database. Tumor regions of interest (ROIs) were meticulously outlined by an experienced thoracic surgeon utilizing 3D Slicer software, followed by validation and refinement by a senior clinical expert to ensure the accuracy of tumor localization and the extraction of radiomic features (**Figure 9B**). A total of 129 radiomic features were extracted from each patient sample (Supplementary File S8). Initial Pearson correlation analysis revealed seven features that exhibited significant correlations with the combined expression of PSMB5, PSMB7, and SLC16A3. Dimensionality reduction through LASSO regression (**Figures 9C, D**) resulted in a final linear regression model that included three critical radiomic features (Supplementary File S9). Based on the combined expression levels of the three key genes, patients were stratified into a high-expression group (n=9) and a low-expression group (n=18). The model displayed robust discriminative capacity, as evidenced by AUC of 0.765, indicating strong predictive performance (**Figure 9E**). Additionally, a strong positive correlation was observed between the LASSO model predictions and the actual combined expression levels of the three genes (**Figure 9F**), further supporting the concordance between radiomic features and molecular expression patterns. In summary, this research lays the groundwork for a preliminary CT radiomics model that facilitates noninvasive evaluation of immunoproteasome subunits (PSMB5, PSMB7) and the metabolic transporter SLC16A3 in LUAD. To assess the reliability of this model, a rigorous 3-fold cross-validation was implemented (**Supplementary Figures 8A, B**), which yielded a favorable overall predictive performance with an AUC of 0.759 and a significant correlation at P<0.05, while effectively mitigating the risk of overfitting. Across the entire cross-validation workflow, ClusterProminence and Complexity emerged as the two consistently identified core radiomic features. This model has the potential to predict preoperatively the activity of tumor immunoproteasomes and the status of metabolic reprogramming within the tumor microenvironment. Additionally, it may function as an imaging biomarker to inform personalized treatment strategies, thus contributing to the progression of noninvasive precision diagnostics in lung cancer.

## Discussion

This research integrates single-cell transcriptomics and radiomics to explore key molecules associated with lung adenocarcinoma metastasis systematically and correlates their expression with CT imaging features. Moreover, it constructs a non-invasive predictive model based on CT radiomics. Our findings suggest that the proteasome core subunits (PSMB5, PSMB7) and the lactate transporter (SLC16A3) may form a synergistic network, implicating their potential role in metastasis. The model facilitates a preliminary radiomic assessment of the expression patterns of these molecules, thereby offering a novel approach for preoperative metastasis risk prediction and individualized treatment.

Utilizing an analysis of single-cell data, this investigation delineated the evolutionary pathway of malignant epithelial cells in lung adenocarcinoma, tracing their progression from primary tumors to metastatic stages. A specific metastasis-associated subpopulation of cells, designated as Cluster 1, was found to be significantly enriched in metastatic samples and correlated with unfavorable prognostic outcomes. This observation is consistent with the prevailing hypothesis regarding the influence of tumor heterogeneity on the metastatic process (30). Additionally, a further examination employing WGCNA revealed a co-expression module (Module 1) that exhibited a notable correlation with this subpopulation. Comprising principal genes involved in protein degradation (PSMB5, PSMB7) and metabolic transport (SLC16A3), this module showed higher expression levels in metastatic tumor cells. These results imply that these functionally distinct molecules may interact spatially and transcriptionally to collaboratively modulate the metastasis of lung adenocarcinoma.

PSMB5 and PSMB7 are key catalytic subunits of the 20S proteasome. Their overexpression in various cancers has been reported to be associated with accelerated cell proliferation and resistance to proteasome inhibitors (31), which may be linked to enhanced degradation of regulatory factors such as cyclins. SLC16A3 (MCT4), as a lactate transporter, plays an important role in maintaining acid-base balance in glycolytic tumor cells, promoting microenvironment acidification, and driving tumor progression (32–34). Previous studies have largely focused on the independent roles of proteasome function or metabolic reprogramming in cancer (35). This study, through integrated single-cell analysis, revealed that PSMB5, PSMB7 and SLC16A3 exhibit a coordinately upregulated expression pattern during lung adenocarcinoma metastasis. Functional enrichment analysis showed that genes associated with this pattern are significantly enriched in proliferation-related pathways such as cell cycle, E2F targets and MYC signaling, and are also linked to metabolic processes including mTORC1 signaling and oxidative phosphorylation. These results suggest a potential functional interplay between proteasome-mediated proteostasis and lactate transporter-mediated metabolic adaptation, which may jointly support the proliferation and survival of metastatic tumor cells. This provides an integrative perspective for understanding the molecular basis of lung adenocarcinoma metastasis.

To further explore the synergistic regulatory mechanism among PSMB5, PSMB7, and SLC16A3, we performed interaction analysis using the STRING database. PSMB5 and PSMB7 might directly interact, whereas SLC16A3 could be indirectly linked via the transcription factors MYC and HIF-1α. These observations suggest that the three genes may form a regulatory module integrating proteasome function and metabolic reprogramming (**Supplementary Figure 8C**). In line with previous studies, MYC has been reported to positively regulate proteasome subunits including PSMB5 and PSMB7 to maintain protein homeostasis and tumor cell proliferation (36) and is also implicated in metabolic reprogramming (37); HIF-1α may directly activate SLC16A3 under hypoxic conditions, promoting glycolysis and tumor microenvironment acidification (38). Collectively, these findings imply that PSMB5, PSMB7, and SLC16A3 may cooperatively drive LUAD metastasis by forming a regulatory network linked to MYC and HIF-1α, thereby coordinating proteasome activity and metabolic reprogramming.

To translate these findings into clinical applications, we established and validated a CT radiomics-based prediction model (AUC = 0.765) that successfully connects imaging features to the co-expression pattern of these key genes. This model exemplifies the “visual genomics” potential of radiomics, consistent with recent efforts to infer molecular traits from non-invasive imaging (39, 40). While studies have used radiomics to predict EGFR or PD-L1 status in lung cancer (41–43), our model specifically targets the co-expression network of proteasome and metabolic genes in LUAD metastasis. Thereby, it may offer a tool for the non-invasive preoperative assessment of tumor aggressiveness and provides actionable information to guide personalized treatment, especially for patients in whom biopsy is unfeasible or yields insufficient tissue.

This study acknowledges several limitations that warrant consideration. Firstly, the restricted availability of paired single-cell and transcriptomic datasets in accessible public repositories necessitates further validation of our results within larger prospective cohorts to enhance their generalizability. Secondly, while initial validation was conducted through IHC and RT-qPCR, the specific molecular pathways through which PSMB5, PSMB7 and SLC16A3 facilitate metastasis in lung adenocarcinoma have yet to be clarified through direct functional assays both *in vitro* and *in vivo*. Thirdly, the radiomics model was constructed based on a relatively modest sample size (n=27), which poses a potential risk of overfitting. Therefore, its robustness, generalizability, and the interpretability of the relationship between imaging characteristics and biological functions must be rigorously evaluated in independent external cohorts. Lastly, the interpretative capacity linking radiomic features to biological functions remains limited. A significant challenge for forthcoming research will be to more explicitly define the associations among imaging “characteristics-gene expression-biological behavior”.

The two core radiomic features, ClusterProminence and Complexity, are biologically interpretable and aligned with the inferred functions of PSMB5, PSMB7 and SLC16A3, supported by our multi-omics data. ClusterProminence reflects intratumoral structural heterogeneity potentially driven by PSMB5/PSMB7-mediated proteasome hyperactivity, as these genes are enriched in cell cycle and proliferation-related pathways that accelerate clonal expansion, which translates to uneven cell density in CT imaging. Complexity correlates with tumor texture irregularity likely arising from SLC16A3-induced metabolic reprogramming (including TME acidification and glycolysis), where metabolic heterogeneity induces uneven angiogenesis and focal necrosis—features captured as increased texture complexity in imaging. Collectively, the combined contribution of these two features establishes a plausible imaging-molecular link consistent with well-established cancer mechanisms, though direct experimental validation of this association is warranted in future work.

Leveraging the synergistic interactions identified among the three pivotal genes, we performed a drug screening analysis that indicated promising therapeutic approaches aimed at this network, including phosphodiesterase inhibitors and ion exchange inhibitors. Phosphodiesterase inhibitors modulate cyclic adenosine monophosphate/cyclic guanosine monophosphate (cAMP/cGMP) signaling to regulate cell proliferation, metabolism and immune responses (44), and their associated signaling pathways may potentially intersect with PSMB5/PSMB7-mediated proteolytic regulation and SLC16A3-driven metabolic adaptation in LUAD. Ion exchange inhibitors can regulate intracellular ion homeostasis and energy metabolism (45), which might in turn interfere with SLC16A3-dependent lactate efflux and the subsequent acidification of the tumor microenvironment, thus providing a potential new perspective for improving the efficacy of targeted therapy and chemotherapy in LUAD. This offers a fresh viewpoint for addressing resistance to both targeted therapies and chemotherapy in LUAD. Nonetheless, the effectiveness and safety of these potential compounds necessitate additional confirmation through preclinical investigations followed by subsequent clinical trials.

## Conclusion

This research highlights the pivotal role of metastasis in the progression and prognosis of LUAD, an intricate process that remains challenging to predict. By integrating single-cell RNA sequencing with multimodal imaging and bioinformatics, we identified three genes (PSMB5, PSMB7 and SLC16A3) that are consistently upregulated in metastatic lesions and correlate with both poorer survival and imaging phenotypes associated with higher tumor burden. These findings establish a tangible molecular-imaging link and position the identified genes as promising biomarkers for metastatic risk assessment.

## Data Availability

Publicly available datasets were analyzed in this study. These data can be accessed here: [TCGA database (https://portal.gdc.cancer.gov/) and GEO database (https://www.ncbi.nlm.nih.gov/geo/query/acc.cgi, accession numbers: GSE235782, GSE277742, and GSE31210)].

## Supplementary material

Supplementary Figure 1 Single-cell atlas construction and copy number variation analysis of epithelial cells. (A) t-SNE dimensionality reduction visualization showing clustering results of 15 cell subpopulations. (B) Distribution of six major cell types in the t-SNE plot. (C-F) Copy number variation (CNV) analysis of epithelial cells in different lung adenocarcinoma samples, showing CNV scores and inter-sample correlation analysis for each sample: (C) AIS1, (D) AIS2, (E) MT1, (F) MT2.

Supplementary Figure 2 Distribution and comparison of malignant epithelial cells in dimensionality-reduced spaces. **(A)** Comparison of the distribution of malignant epithelial cells and non-malignant cells in the UMAP plot. **(B)** Distinction between malignant epithelial cells and other cells in the t-SNE plot. **(C)** Visual distribution of epithelial cells in the UMAP dimensionality reduction plot. **(D)** Visual distribution of malignant epithelial cells in the UMAP dimensionality reduction plot. **(E)** Distribution of six malignant epithelial cell subpopulations in the t-SNE plot. **(F)** Comparison of the distribution of malignant epithelial cells from primary and metastatic sites in the t-SNE dimensionality reduction plot.

Supplementary Figure 3 Transcriptional dynamics of malignant epithelial cells and cell-cell communication network analysis. **(A)** Heatmap showing changes in key differentially expressed genes along the developmental trajectory in malignant epithelial cells. **(B)** Network diagram of communication frequency between cell types. **(C)** Network diagram of communication strength between cell types. **(D–E)** Chord diagrams illustrating key ligand–receptor interactions of MIF and MK signaling pathways in cell-cell communication.

Supplementary Figure 4 Intercellular communication network of malignant epithelial cells in the LUAD microenvironment. **(A)** Heatmap showing signaling pathways where metastatic malignant epithelial cells act as senders. **(B)** Heatmap showing signaling pathways where metastatic malignant epithelial cells act as receivers. **(C)** Network diagram depicting the interaction frequency among six malignant subclusters and other cell types. **(D)** Network diagram depicting the interaction strength among six malignant subclusters and other cell types. **(E)** Bubble plot illustrates cell-type-specific signaling pathways enriched in the six malignant subclusters.

Supplementary Figure 5 Construction and validation of prognostic risk models based on gene module scores. **(A)** Distribution of Module 1 gene set scores across six malignant epithelial cell subpopulations. **(B)** Distribution of patient risk scores in the TCGA-LUAD cohort. **(C)** Association between patient survival status and risk scores in the TCGA-LUAD cohort. **(D)** Distribution of patient risk scores in the independent validation cohort GSE31210. **(E)** Correspondence between patient survival status and risk scores in the GSE31210 cohort. **(F)** Enrichment analysis results of high-risk group in cell proliferation-related pathways, including E2F target genes, G2M checkpoint and MYC signaling pathway. **(G)** Enrichment analysis results of high-risk group in signal transduction and metabolic regulation pathways, covering mTORC1 and PI3K-AKT-mTOR signaling pathways. (H) Enrichment analysis of high-risk group in tumor microenvironment and metastasis-related pathways, including hypoxia response and DNA repair pathways.

Supplementary Figure 6 Association analysis of key gene expression levels with prognosis in lung adenocarcinoma. **(A–G)** Kaplan-Meier curves of overall survival based on median expression grouping for C4orf48, KRT81, LGALS3, PSMB5, PSMB7, S100P and SLC16A3 expression levels. **Two-sided log-rank test and univariate Cox regression.

Supplementary Figure 7 Functional enrichment analysis of genes related to PSMB5, PSMB7, and SLC16A3. **(A)** Gene Ontology (GO) functional enrichment analysis results of the related genes. **(B)** KEGG pathway enrichment analysis results of the related genes.

Supplementary Figure 8 Prognostic validation and regulatory network of the PSMB5+PSMB7+SLC16A3 signature in LUAD. **(A)** Three-fold cross-validation ROC curve of the PSMB5+PSMB7+SLC16A3 expression signature for outcome prediction. **(B)** Correlation between the PSMB5+PSMB7+SLC16A3 expression signature and LASSO model fitted values (three-fold cross-validation, R = 0.454, p = 0.017, n = 27). **(C)** Protein-protein interaction network of PSMB5, PSMB7, SLC16A3 and their key co-regulators. **Statistical significance was assessed using the two-sided DeLong test for ROC comparisons and the two-sided Pearson correlation test for association analyses.
