# Supplementary material for: Noninvasive assessment of core metastatic genes in lung adenocarcinoma: development of a predictive model integrating single-cell transcriptomics and radiomics
Source: Front Oncol. 2026 Mar 16;16:1784914. doi: 10.3389/fonc.2026.1784914 (PMC13033551; doi:10.3389/fonc.2026.1784914)
Supplement: Supplementary file 9 [file Table1.docx]

Table S1. Antibody of PSMB5, PSMB7 and SLC16A3.

| PSMB5 | proteintech | 19178-1-AP |
| --- | --- | --- |
| PSMB7 | proteintech | 30283-1-AP |
| SLC16A3 | proteintech | 22787-1-AP |
