# Supplementary material for: Noninvasive assessment of core metastatic genes in lung adenocarcinoma: development of a predictive model integrating single-cell transcriptomics and radiomics
Source: Front Oncol. 2026 Mar 16;16:1784914. doi: 10.3389/fonc.2026.1784914 (PMC13033551; doi:10.3389/fonc.2026.1784914)
Supplement: Supplementary file 10 [file Table2.docx]

Table S2. The sequences of primers of PSMB5, PSMB7 and SLC16A3.

| PSMB5 | Forward Primer | AGGAACGCATCTCTGTAGCAG |
| --- | --- | --- |
|  | Reverse Primer | AGGGCCTCTCTTATCCCAGC |
| PSMB7 | Forward Primer | TTTCTCCGCCCATACACAGTG |
|  | Reverse Primer | AGCACCTCAATCTCCAGAGGA |
| SLC16A3 | Forward Primer | CCATGCTCTACGGGACAGG |
|  | Reverse Primer | GCTTGCTGAAGTAGCGGTT |
